# Rethinking sex effects in schizophrenia: continuous brain sex-scores and their clinical correlates

**DOI:** 10.1192/j.eurpsy.2026.10630

**Published:** 2026-07-31

**Authors:** N. Hostalet, D. Vosberg, C. Almodóvar-Payá, M. Latorre-Guardia, M. Giralt-López, A. Guerrero-Pedraza, S. Sarró, R. Salvador, E. Pomarol-Clotet, T. Paus, M. Fatjó-Vilas

**Affiliations:** 1 FIDMAG Germanes Hospitalàries Research Foundation; 2 Departament de Biologia Evolutiva, Ecologia i Ciències Ambientals, Facultat de Biologia, Universitat de Barcelona, Barcelona; 3 CIBERSAM, Instituto de Salud Carlos III, Madrid, Spain; 4 Centre de Recherche du CHU Sainte-Justine; 5 Departament of Neuroscience, University of Montreal, Montreal, Canada; 6 Servei de Psiquiatria Infantil i de l’Adolescència, Hospital Universitari Germans Trias i Pujol, Badalona; 7 Departament de Psiquiatria i Medicina legal, Universitat Autònoma de Barcelona, Barcelona; 8 Institut Recerca Germans Trias i Pujol, Badalona; 9 Fundación Hospitalarias Sant Boi, Sant Boi, Spain; 10 Department of Psychiatry and Addictology, University of Montreal, University of Montreal, Montreal, Canada

## Abstract

**Introduction:**

Sex differences in schizophrenia (SZ) are well-documented in terms of age of onset, symptoms, and treatment response. However, sex is often treated as a covariate rather than a primary variable of interest. Continuous “sex-scores” have been proposed to quantify the “maleness/femaleness” of multiple traits, as a complement to biological sex.

**Objectives:**

We aimed: i) to compute brain sex-scores in healthy individuals (HC) and SZ patients and; ii) to examine the association of brain sex-scores with SZ symptoms.

**Methods:**

In Sample A (215 males, 211 females HC), sex effect coefficients were obtained for 34 cortical regions (Desikan-Killiany atlas) through linear regressions of cortical thickness (CT) and surface area (SA). In Sample B (237 SZ patients), individual-level CT and SA values were summed and weighted by the sex effect coefficients obtained from Sample A to compute sex-scores ranging from 0 (maximum maleness) to 1 (maximum femaleness). We assessed correlations between sex-scores and Positive and Negative Syndrome Scale (PANSS) in SZ patients, separately for males and females.

**Results:**

CT sex-scores showed significant correlations with PANSS-Negative symptoms in females (r^2^ =-0.4; p_FDR_=0.017). In males, CT sex-scores were associated with PANSS-Positive symptoms (r^2^=-0.18; p_FDR_=0.033) and general psychopathology (r^2^=-0.24; p_FDR_=0.009).

**Conclusions:**

Our data suggest that lower CT sex-scores (associated with greater maleness) are related to more severe symptoms, highlighting the importance of considering sex-related variability in a quantitative manner. Then, sex-scores emerge as a useful tool to study sex- and gender-related variability in the neurobiology and clinical expression of schizophrenia.

**Acknowledgements:**

ISCIII project-PI20/01002, grant-FI21/00093-NH, grant-CP20/00072-MFV co-funded by ERDF/ESF; AGAUR-2021SGR01475; Beca Santander-Estades formatives Espanya i estranger 2024 doctorands UB-NH; Beca de Recerca Bàsica de l’Acadèmia 2025.

**Disclosure of Interest:**

None Declared

